# Differences in Physical Fitness According to Nutritional Status Among Rural Schoolchildren

**DOI:** 10.3390/jfmk10040364

**Published:** 2025-09-24

**Authors:** Miguel Alarcón-Rivera, María Gracia Jélvez Correa, Nayareth González Parada, Sebastián Aldana Rosales, Felipe Montecino-Rojas, Pablo Luna-Villouta, Exal Garcia-Carrillo, Héctor Fuentes-Barría, Raúl Aguilera-Eguía, Lissé Angarita-Davila

**Affiliations:** 1 Escuela de Enfermería, Facultad de Salud, Universidad Santo Tomás, Talca 3460000, Chile; m.jelvez4@alumnos.santotomas.cl (M.G.J.C.); n.gonzalez141@alumnos.santotomas.cl (N.G.P.); s.aldana@alumnos.santotomas.cl (S.A.R.); 2 Escuela de Ciencias del Deporte y Actividad Física, Facultad de Salud, Universidad Santo Tomás, Talca 3460000, Chile; felipecienciasdeldeporte@gmail.com; 3 Departamento de Educación Física, Facultad de Educación, Universidad de Concepción, Concepción 4070371, Chile; pabloluna@udec.cl; 4 Department of Physical Activity Sciences, Faculty of Education Sciences, Universidad Católica del Maule, Talca 3460000, Chile; exal.garcia@gmail.com; 5 Department of Physical Activity Sciences, Universidad de Los Lagos, Osorno 5290000, Chile; 6 Vicerrectoría de Investigación e Innovación, Universidad Arturo Prat, Iquique 1100000, Chile; 7 Escuela de Odontología, Facultad de Odontología, Universidad Andres Bello, Concepción 3349001, Chile; 8 Departamento de Salud Pública, Facultad de Medicina, Universidad Católica de la Santísima Concepción, Concepción 3349001, Chile; raguilerae@ucsc.cl; 9 Escuela de Nutrición y Dietética, Facultad de Medicina, Universidad Andres Bello, Concepción 3349001, Chile; lisse.angarita@unab.cl

**Keywords:** agility, body mass index, cardiovascular endurance, motor activity, motor skills, muscular strength, nutritional status, obesity, physical fitness

## Abstract

**Background:** Childhood overweight and obesity are increasing public health concerns globally, with a high prevalence in Chile, particularly in rural areas. Excess weight may impair physical fitness, affecting children’s overall health and development. **Objectives:** This study aimed to compare the physical fitness of schoolchildren with normal weight (NW) and overweight/obesity (OW/OB) from a rural school in Maule, Chile. **Methods:** A total of 87 students (boys and girls, aged 9–14 years) were evaluated and classified into NW and OW/OB groups based on their body mass index. Physical fitness was assessed using the 6 min walk test (6MWT) for cardiovascular endurance, handgrip strength (HGS) and squat jump (SJ) for muscular strength, 20 m sprint for speed, and 4 × 10 m shuttle run for agility. **Results:** Significant differences were found between groups in most physical fitness components. The NW group covered 11.13% more distance in the 6MWT than the OW/OB group (*p* < 0.001; d = 1.28). NW children also performed better in the 20 m sprint (*p* = 0.023; d = 1.02) and the 4 × 10 m shuttle run (*p* < 0.001; d = 0.72). SJ was higher in the NW group (*p* = 0.004; d = 0.45). No significant differences were found in HGS (*p* = 0.893; d = 0.01). **Conclusions:** Children with normal weight demonstrated better physical fitness compared to their overweight or obese peers. These findings support the need for targeted strategies to prevent overweight and obesity in rural schoolchildren to improve physical health and functional capacities.

## 1. Introduction

According to data from the World Obesity Federation, the global prevalence of obesity among children and adolescents (aged 5–19 years) has been estimated at 206 million by 2025 and 254 million by 2030 [1]. In Chile, at the school level, as early as 2019 it was reported that all educational levels (pre-kindergarten, kindergarten, 1st grade, 5th grade, and 9th grade) had an obesity prevalence of 23%, with 6% corresponding to severe obesity [2,3]. In the pediatric population, overweight and obesity combined reached 54%, which translated into an increase of 2 percentage points in total obesity and 1.2% in severe obesity [2,3]. Even more concerning is that around 3 out of 5 fifth-grade students were overweight or obese. [2,3]. Specifically in the Maule region, a study reported that among a sample of students from four educational institutions, 36.1% were classified as overweight and 27.5% as obese, highlighting this issue as a significant local public health changed to concern [4].

A low-cost and accessible method for assessing body composition imbalances and classifying schoolchildren according to their nutritional status is the Body Mass Index (BMI), which is widely used for evaluating nutritional status in school-aged populations [5]. This indicator correlates strongly with nutritional status in adolescents and appears to have predictive value for the risk of cardiometabolic complications [6]. BMI classifies nutritional status into underweight, normal weight, overweight, pre-obesity, obesity, type I obesity, type II obesity, and type III obesity [7]. Obesity, considered a chronic disease, is characterized by the excessive accumulation of body fat, which increases the risk of conditions such as diabetes mellitus and cardiovascular diseases [8]. In fact, being overweight or obese is considered a predictor of poor health outcomes, as well as reduced physical fitness and motor coordination in school-aged children [9,10].

Physical fitness is defined as the condition of an individual, determined by their levels of cardiovascular endurance, muscular strength, speed, and agility [11]. These physical attributes are key components of fitness and are directly related to the functioning of the cardiovascular and respiratory systems [12]. Physical fitness can be negatively affected by excess weight, particularly due to imbalanced body composition, as the accumulation of fat mass reduces cardiovascular efficiency, limits mobility, and impairs motor function in school-aged children [13,14]. Moreover, overweight and obesity are closely associated with health problems such as high blood pressure, diabetes mellitus, abdominal obesity, and other metabolic disorders that adversely affect the development of these physical attributes [15].

In this context, 37% of children and adolescents (5–17 years old) reported engaging in at least 60 min of physical activity on four or more days per week [16]. Similar patterns emerge when comparing urban and rural settings, where both contexts show that approximately 90% of participants report accumulating less than seven hours of physical activity per week [17]. Coronado Vázquez et al., [18] indicates the existence of an impact on the rural population (<5000 inhabitants). Specifically, rural areas showed a prevalence of 6.84% for overweight and 5.83% for obesity. Moreover, among schoolchildren aged 6, 11, and 14 years living in these small municipalities, the risk of obesity was 1.49 times higher (OR = 1.49; 95% CI: 1.13–1.95) and the risk of overweight was 1.33 times higher (OR = 1.33; 95% CI: 1.06–1.67), highlighting the greater vulnerability of children in rural settings. [18]. Given the rising global prevalence of overweight and obesity in children, particularly in rural populations—and their impact on physical fitness, it is essential to assess differences in physical fitness between normal-weight and overweight/obese schoolchildren. However, limited research has addressed how nutritional status influences physical fitness among rural schoolchildren in Chile, despite the high prevalence of over-weight and obesity reported in these communities This analysis helps to better under-stand how these conditions affect overall health and the development of physical attributes.

Therefore, the aim of this study is to compare physical fitness between normal-weight and overweight/obese children in a rural school in the commune of Maule, Chile.

## 2. Materials and Methods

### 2.1. Study Design

This study employed cross-sectional, observational, and comparative design. The sample consisted of 87 schoolchildren of both sexes from a school located in the commune of Maule, Chile. A non-probabilistic, convenience sampling method was used.

The study adhered to the principles outlined in the Declaration of Helsinki for re-search involving human subjects [19]. Written informed consent was obtained from the parents or legal guardians of all participants. Additionally, all schoolchildren are provided with written informed assent. The study protocol was approved by the Ethics Committee of University Santo Tomás (Code: 23-1, Date: 9 August 2023). This is in line with the “Strengthening the Reporting of Observational Studies in Epidemiology” [20].

### 2.2. Context

Childhood overweight and obesity are growing public health concerns globally, associated with increased risk of chronic diseases and impaired physical fitness. Understanding the physical fitness differences between normal-weight and over-weight/obese children is essential to design effective interventions promoting health and preventing morbidity [3,21,22]. However, there is limited evidence regarding the specific physical fitness profiles of school-aged children in the Maule region of Chile, especially using validated fitness assessments adapted to this population. This cross-sectional observational study aims to compare physical fitness parameters between normal-weight and overweight/obese schoolchildren, providing valuable data to inform public health strategies and contribute to the regional and national under-standing of childhood obesity impacts on functional health.

### 2.3. Participants

The following eligibility criteria were defined: inclusion criteria (a) male and female students, (b) aged between 9 and 14 years, (c) students officially enrolled and regularly attending classes in accordance with the guidelines of the Chilean Ministry of Education. Exclusion criteria were defined as (a) having any musculoskeletal condition that prevents participation in the tests, (b) student does not show willingness to perform the test with the required intensity, (c) student classified as underweight. After applying the eligibility criteria and assessing nutritional status, the schoolchildren were divided into two groups: 47 normal-weight (NW) and 40 overweight/obese (OW/OB).

### 2.4. Data Collection Process

A cohort of schoolchildren was evaluated between July and August 2024, as part of a cross-sectional study developed within the framework of a collaboration agreement between a school in the commune of Maule and the School of Nursing at University Santo Tomás. The process began with the signing of informed consent from parents or legal guardians and assent by the schoolchildren. The evaluation began early in the morning and continued throughout the day until the end of the day (approximately 8:00–17:00 h).

First, weight and height were measured to calculate BMI and classify participants. Subsequently, physical fitness was assessed. After this measure, a general and specific warm-up was carried out under the supervision of a sports coach. This consisted of 10 min of continuous running at a light pace, followed by active stretching progressively involving the main muscle groups, from the lower to the upper body. Once the warm-up was completed, the physical test battery began. Body strength was first assessed through the squat jump (SJ). Then, linear speed was measured with the 20 m sprint test, followed by agility using the 4 × 10 m shuttle run test. Finally, the session concluded with the six-minute walk test (6MWT). Some of the fitness tests used in this study were based on the PRE-FIT battery [23], which is validated for children; however, not all components of the original battery were included due to logistical constraints and the specific objectives of the study.

### 2.5. Instruments

To divide the study groups, nutritional status was quantified using the BMI. First, body weight was measured using a Seca^®^ scale (Hamburg, Germany; accuracy of 0.1 kg). Participants were asked to step onto the scale wearing light clothing and barefoot, standing upright with arms at their sides. Then, height was measured using a Seca^®^ stadiometer (Hamburg, Germany; accuracy of 0.1 mm). Both instruments meet the International Society for the Advancement of Kinanthropometry (ISAK) recommendations for accuracy and measurement range [24].

Participants were asked to stand barefoot and look straight ahead. It was ensured that the shoulders were level, arms relaxed, and that the head, shoulders, and buttocks were in contact with the backboard. With these measurements, BMI was calculated using the following equation: BMI = weight (kg)/height (m^2^). Based on the result, participants were classified as underweight, normal weight, overweight, or obese [25].

#### 2.5.1. Cardiorespiratory Endurance

To assess endurance, the 6MWT was applied, which consists of walking the greatest possible distance in a period of six minutes [26]. First, the perimeter of the school’s sports field was measured using a Truper odometer (model 15831), resulting in a perimeter of 82 m. Participants were then asked to complete as many laps around the field as possible. At the end of the test, participants were instructed to remain in place while an evaluator measured the distance from the starting point of the last lap to the point reached. The total distance covered (in meters) was obtained by multiplying the number of complete laps by the 82 m perimeter of the field and adding the final partial distance.

#### 2.5.2. Muscle Strength

To assess lower limb muscular strength, the vertical jump test (SJ) was used. A Chronojump Boscosystem^®^ jump mat (DINA2, Barcelona, Spain) was employed for this purpose. The test consisted of asking the student to step onto the mat, place their hands on their hips, position their feet shoulder-width apart, and perform a triple flexion of the lower limbs until reaching approximately 90° at the knee and hip joints. The student was instructed to hold this position isometrically for 3 s and then jump vertically, aiming to reach the highest possible height in centimeters. Two attempts were performed, with the best result recorded [27]. Upper limb strength was assessed using handgrip strength (HGS), measured with a Camry^®^ digital dynamometer (EH101; accuracy 0.1 kg, Zhongshan Camry Electronic Co. Ltd., Zhongshan, China). It has demonstrated a high interclass correlation coefficient (ICC = 0.97) when compared to the JAMAR^®^ J00105 hydraulic dynamometer (Bolingbrook, IL, USA) [28]. The students were asked to sit upright, with their dominant arm flexed at 90°, holding the dynamometer without applying any initial force. The dominant arm was simply determined by self-report. Then, at the evaluator’s signal, the participant exerted maximal isometric grip force for 3 s. Three trials were performed with one-minute rest intervals, and the highest value (in kilograms) was recorded for analysis [29].

#### 2.5.3. Speed and Agility

To assess 20 m linear speed, the school’s court was used, which was a flat and even surface with synthetic flooring. A straight 20 m corridor was marked with cones to indicate the start and finish positions. The exact distance was verified using a Truper odometer (model 15831) (Truper, Naucalpan, Mexico). The time was recorded in two attempts using a Casio stopwatch (Casio Sports Stop Watch HS-50W, Casio, Tokyo, Japan), and the best result in seconds was considered for analysis [30]. Agility was evaluated using the 4 × 10 m test, which consists of covering a total of 40 m by running back and forth over a 10 m section four times at maximum speed. The 10 m straight line was marked with cones on a flat, uniform surface with synthetic flooring, and the distance was verified with the Truper odometer (model 15831). At the start of the test, the beginning and end times were recorded with the same Casio stopwatch, and the total time in seconds was used for analysis [31].

### 2.6. Sample Size and Statistical Power

The study included a sample of 87 participants, divided into two groups: 47 nor-mal-weight individuals and 40 overweight/obese individuals. Participants were selected through non-probability convenience sampling based on predefined inclusion criteria. Although the sample size was limited due to logistical and ethical constraints, a post hoc two-sided power analysis was performed using G*Power software version 3.1.9.7 for Windows (Dusseldorf, Germany), with a significance level of 0.05 [32]. Based on the effect sizes observed for the fitness variables, the estimated sample size needed to achieve 80% statistical power was approximately 156 participants. Therefore, the available sample was insufficient to detect smaller effect sizes, such as those observed for the 20 m sprint and 6 min walk tests, with adequate power. However, for variables with larger effect sizes, such as agility and squat jump, the sample size was sufficient. Despite this limitation, the study design and the homogeneity of the base group (*p* > 0.05) minimized variability and preserved internal validity, allowing for accurate interpretation of the results.

### 2.7. Potential Bias

This cross-sectional, observational study employed a non-probabilistic convenience sampling method, which may introduce selection bias, limiting the representatives of the sample and generalizability of the findings to the broader population of schoolchildren [33]. The absence of randomization and control over confounding variables, such as socioeconomic status, dietary habits, or physical activity outside the school setting, could contribute to residual confounding, affecting the observed association [34]. Additionally, some fitness tests were adapted or partially applied due to logistical constraints, which might have affected the completeness and comparability of the physical fitness assessment. The reliance on voluntary participation and assent may have introduced participation bias, as those more motivated or with higher physical fitness levels could be overrepresented [33,34]. Finally, the limited sample size, determined by logistical and ethical factors, reduces statistical power to detect small or moderate effects, which could lead to type II errors. Despite these limitations, the standardized protocols, ethical rigor, and homogeneity of groups at baseline (*p* > 0.05) helped to minimize variability and support the internal validity of the study.

### 2.8. Statistical Analysis

The statistical analysis was performed using GraphPad Prism version 9 (GraphPad Software, San Diego, CA, USA). Descriptive statistics such as mean, standard deviation, minimum, and maximum values were used. Results are presented as mean (X), standard deviation (SD), minimum (MIN) and maximum (MAX). The Kolmogorov–Smirnov test was applied to assess normality, given that the sample included more than 50 participants [35]. The Student *t*-test for independent samples was used to compare parametric results in the 6MWT variables, 20 m linear speed, 4 × 10 m agility and handgrip strength, and the Mann–Whitney U test was applied to compare non-parametric results in the squat jump variable [36,37]. Homogeneity of variances was assessed using Levene’s test, and appropriate adjustments were made when the assumption of equal variances was violated [38]. Statistical significance was set at *p* < 0.05. Cohen’s d was used to quantify the effect size, interpreted as follows: <0.20 = trivial effect; 0.21–0.49 = small effect; 0.50–0.79 = moderate effect; >0.80 = large effect [39].

## 3. Results

Table 1 presents the general characteristics of the sample compared using Student’s *T* test. The mean age was similar between groups, with 12.26 ± 1.19 years for the NW group and 12.23 ± 1.03 years for the OW/OB group, showing no statistically significant difference (*p* = 0.90) and a trivial effect size (d = 0.03), indicating baseline homogeneity in age. Regarding body weight and BMI, significant differences were observed between groups (*p* = 0.001), with large effect sizes (d = 1.83 and 2.63, respectively), reflecting the clear distinction in nutritional status used for group classification. Height showed a small, non-significant difference (*p* = 0.17; d = 0.30). This profile indicates that while groups were comparable in terms of age and height, they were heterogeneous in body weight and BMI, as expected. This baseline homogeneity in key demographic variables (age) alongside clear differences in nutritional measures supports the internal validity of comparisons made between groups.

Regarding the comparisons between groups, significant differences were observed in the 6MWT (*p* < 0.001, d = 1.28), 20 m linear sprint (*p* = 0.023, d = 1.02), 4 × 10 m shuttle run (*p* < 0.001, d = 0.72), and SJ (*p* < 0.004, d = 0.83). Conversely, no significant differences were found between groups for HGS (*p* = 0.893, d = 0.01). These results are presented in Table 2.

## 4. Discussion

The main findings of this study demonstrate significant differences in most physical fitness tests between the NW and OW/OB groups. Specifically, endurance, as measured by the six-minute walk test (6MWT), was greater in the NW group, with a large effect size (d = 1.43). These results are consistent with prior research indicating superior cardiorespiratory fitness in normal-weight children compared to their overweight and obese counterparts. For instance, Renugadevi and Vinod [40], reported that NW schoolchildren (n = 75) covered 6.16% more distance than overweight peers during the 6MWT, with the difference being statistically significant (*p* ≤ 0.01). Similarly, the study by Morinder et al. [41], reported that the distance covered by a group of overweight children was 571 m, while the normal-weight group reached 663 m (*p* < 0.001). This aligns with the results of our study, where the NW group covered 898 m and the OW/OB group 720 m. These differences in favor of normal-weight schoolchildren may be attributed to a higher maximal oxygen consumption (VO_2_ max), which leads to more efficient oxygen uptake and utilization [42]. Additionally, it has been shown that overweight or obese children have reduced mitochondrial function compared to normal-weight peers, which may contribute to the development of conditions such as insulin resistance [43,44,45,46].

Regarding upper-body muscle strength, no significant differences were found between the NW and OW/OB groups (*p* = 0.893). Despite these results, the existing scientific evidence remains controversial. For instance, a study conducted on Spanish schoolchildren evaluated 203 girls and 220 boys, reporting no significant differences in HGS in the left hand (*p* = 0.955) or right hand (*p* = 0.771) between normal-weight and overweight boys. In contrast, significant differences were found among girls for both left-hand (*p* = 0.001) and right-hand (*p* = 0.002) grip strength [46]. In the context of Chilean schoolchildren, Palacio-Agüero et al. [21], assessed relative HGS to body weight and reported significant differences between normal-weight and obese children (*p* ≤ 0.01), as well as between children with and without abdominal obesity (*p* ≤ 0.01). Unlike previous studies, the sample in Palacio-Agüero et al. [21], included approximately 250 participants per sex, which may suggest that a larger sample size allows for the detection of differences across both sexes. In our case, HGS did not show significant differences between groups. These findings may be explained by the fact that individuals with higher fat mass tend to exhibit greater absolute maximal strength, as adiposity acts as an overload stimulus that promotes musculoskeletal adaptations, resulting in increased strength [47,48]. However, when strength is adjusted for body weight (i.e., relative strength), the results tend to favor individuals with normal weight or unaltered body composition [48].

Regarding lower-body muscular strength, our findings indicate significant differences in SJ height (*p* = 0.004, d = 0.45), favoring NW over OW/OB schoolchildren. Simi-lar results were reported by Cowley et al. [49], in a sample of 39 schoolchildren, showing significantly higher jump heights (*p* ≤ 0.01) among NW children. Additionally, the authors noted that other mechanical variables of the jump, such as range of motion and angular velocities, were altered in children with overweight, which may help ex-plain the observed differences. In the same vein, a study conducted on 478 Chilean schoolchildren reported that NW participants achieved greater distances in the standing long jump (*p* = 0.022), supporting the notion that the differences identified in our study may extend across various jump tests [50]. Existing evidence suggests that these differences in jump performance between NW and OW/OB children are mediated by both biomechanical and neural factors, as children with obesity have been shown to exhibit delayed muscle activation in lower-limb muscles, which may result in impaired force production compared to their NW peers [49,51].

In our study, agility performance was also better among NW schoolchildren compared to their OW/OB peers. Previous reports support these findings. Specifically, Ceschia et al. [52], reported significant differences in agility performance (*p* ≤ 0.05) in favor of NW children aged 9 and 10 years. Similarly, the study by Martínez et al. [53], found significant differences (*p* ≤ 0.01) in agility among children aged 6 to 9 years, also favoring those with normal weight. Agility, like speed, is a physical quality with a high technical component. Therefore, the findings of Bowser and Roles [54], which highlight altered gait biomechanics in overweight children, can also be extrapolated to agility performance. Additionally, it has been reported that OW/OB schoolchildren exhibit lower functional movement quality, which is inversely associated with physical fitness [55].

## 5. Clinical and Practical Implications

The findings of this study highlight the urgent need to implement targeted interventions in rural school settings to prevent and reduce overweight and obesity among children. Since nutritional status significantly impacts key components of physical fitness—such as endurance, lower-limb strength, speed, agility, and motor skills—school-based programs should incorporate regular, structured physical activity that focuses on both aerobic and strength training, as well as activities that enhance fundamental motor skills [56,57,58]. Additionally, strategies to promote healthy eating habits and reduce sedentary behaviors should be integrated into the school curriculum and supported by family and community engagement [59,60]. These measures may not only improve physical performance but also contribute to better long-term health outcomes and reduce the risk of chronic diseases later in life [61].

## 6. Limitations and Future Directions

This study has several limitations. First, the cross-sectional design prevents establishing causal relationships between nutritional status and physical fitness. Second, the use of non-probabilistic convenience sampling limits the generalizability of the results to other rural or urban populations. Third, the relatively small sample size reduced the statistical power for detecting small or moderate differences in some variables, such as handgrip strength [33,34,62]. Additionally, certain potential confounders, such as socioeconomic status, dietary intake, extracurricular physical activity, and level of familiarity with the tests performed, were not controlled for and could have influenced the results in rural context [63,64]. Future research should employ larger, randomly selected samples, consider longitudinal designs to assess causality [33,34], and incorporate a more comprehensive set of fitness tests alongside lifestyle and environmental variables. In this context, it is crucial to consider BMI as a complementary measure rather than a primary metric due to its well-documented limitations [65].

## 7. Conclusions

The results of this study demonstrate significant differences in lower-limb muscular strength, as well as in endurance, speed, and agility, favoring schoolchildren with an adequate nutritional status. In contrast, no significant differences were observed between groups in upper-limb muscular strength. Nevertheless, schoolchildren with a healthy body composition exhibited better overall physical fitness compared to their peers with overweight or obesity.

## Figures and Tables

**Table 1:** Characteristics of the sample.

Variables	NW Group (n = 47)			OW/OB Group (n = 40)				
	MIN	MAX	X ± SD	MIN	MAX	X ± SD	*p*-Value	Effect Size
Age (years)	10.11	14.4	12.26 ± 1.19	10.12	14.2	12.23 ± 1.03	0.90	0.03 (trivial)
Body weight (kg)	35.4	75.8	51.19 ± 7.71	46.4	101.7	71.37 ± 13.59	<0.001 **	1.83 (large)
Height (cm)	135	176	153.32 ± 8.83	134	177	155.92 ± 8.47	0.17	0.30 (small)
BMI (kg/m^2^)	18.02	24.88	21.71 ± 2	25.35	36.35	29.34 ± 3.58	<0.001 **	2.63 (large)

MIN: minimum, MAX: maximum, X: mean, SD: standard deviation; BMI: Body weight mass index; NW: Normal weight; OW/OB: overweight/obese. **: *p* value < 0.001.

**Table 2:** Differences in the physical fitness between normal weight and overweight/obese schoolchildren (n = 87).

Variable	Group	X ± SD	*p*-Value	Effect Size
6MWT (m)	NW	898.99 ± 158.05	<0.001 **	1.28 (*large*)
	OW/OB	720.29 ± 116.92		
20 m sprint (s)	NW	4.22 ± 0.49	0.023 *	1.02 (large)
	OW/OB	4.90 ± 0.80		
4 × 10 m agility (s)	NW	12.35 ± 1.03	<0.001 **	0.72 (Moderate)
	OW/OB	13.23 ± 1.40		
SJ (CM)	NW	15.45 ± 4.50	0.004 *	0.45 (small)
	OW/OB	13.42 ± 4.56		
HGS (kg)	NW	23.72 ± 6.56	0.893	0.01 (*trivial*)
	OW/OB	23.65 ± 6.17		

*: *p* value < 0.05; **: *p* value < 0.001.

## Data Availability

The datasets used and/or analyzed during the current study are available from the corresponding author upon reasonable request.

## Abbreviations

The following abbreviations are used in this manuscript:

BMI	Body Mass Index
NW	Normal-Weight
OW/OB	Overweight/Obese
SJ	Squat Jump
